# History and Application of Mechanical Assist Devices as a Bridge to Heart Transplant: A Review and Perspectives in Brazil

**DOI:** 10.21470/1678-9741-2025-0906

**Published:** 2025-08-13

**Authors:** Alvaro Perazzo, Fabio Antônio Gaiotto, Samuel Padovani Steffen, Shirlyne Fabianni Dias Gaspar, Vanessa Simioni Faria, Rebeca Cavalcante Silva Ferreira, Aline Carbonera, Danielle Louvet Guazzelli, Jael Andrea Rioja Gamboa, Leonardo Flud Ideal, Helen Lima Gomes, Alexandre Targino Gomes Falcão Filho, Andre Loureiro Fernandes, Luiza Hermanny Campos, Camila Lambert Steffen, Carolina Limongi, Renato Leal Varjão, Monica Samuel Avila, Luis Fernando Bernal da Costa Seguro, Sandrigo Mangini, Fabiana Goulart Marcondes-Braga, Gabriel Barros Aulicino, Iascara Wozniak de Campos, Ronaldo Honorato Barros Santos, Domingos Dias Lourenço Filho, Mariusz Kowalewski, Daniele Ronco, Matteo Matteucci, Paolo Meani, Claudio Francesco Russo, Michele di Mauro, Silvia Mariani, Antonio Loforte, Dominik Wiedmann, Fernando Bacal, Glenn Whitman, Prakash Punjabi, Roberto Lorusso, Fabio B Jatene

**Affiliations:** 1 Núcleo de Transplante do Instituto do Coração do Hospital das Clínicas da Faculdade de Medicina da Universidade de São Paulo, São Paulo, AP - Brazil; 2 Departamento de Cirurgia Cardiovascular do Instituto do Coração do Hospital das Clínicas da Faculdade de Medicina da Universidade de São Paulo, São Paulo, SP - Brazil; 3 Cardio-Thoracic Surgery Department, Heart and Vascular Centre, Maastricht University Medical Centre (MUMC), Cardiovascular Research Centre Maastricht (CARIM), Maastricht - The Netherlands; 4 Faculdade Israelita de Ciências da Saúde Albert Einstein (FICSAE), São Paulo, SP - Brazil; 5 Departamento de Cirurgia Cardiovascular, Santa Casa de Curitiba, Curitiba, PR - Brazil; 6 Department of Cardiac Surgery and Transplantology, National Medical Institute of the Ministry of Interior and Administration, Varsóvia - Poland; 7 Cardiac Surgery Unit, Fondazione IRCCS San Gerardo dei Tintori, Monza - Italy; 8 Cardiac Surgery and Heart Transplant Unit, Cardiac Thoracic and Vascular Department, Niguarda Hospital, Milão - Italy; 9 Cardiothoracic and vascular Anestesia and Intensive Care, ASST Grande Ospedale Metropolitano, Niguarda, Milão - Italy; 10 Mechanical Circulatory Support Program, University of Turin, Turin - Italy; 11 Cardiac Surgery and Mechanical Circulatory Support Department, University of Wien, Wien - Austria; 12 Cardiovascular Surgery Department, Johns Hopkins School of Medicine Faculty, Baltimore - USA; 13 Cardiothoracic Surgery Department, Imperial College London, National Heart and Lung Institute, Londres - United Kingdom

**Keywords:** Cardiac Surgical Procedures, Postoperative Care, Noninvasive Ventilation, Systematic Review.

## Abstract

Mechanical circulatory support (MCS) devices have evolved significantly over the past decades and play a vital role in managing end-stage heart failure, especially as a bridge to heart transplantation. From the pioneering heart-lung machines to third-generation ventricular assist devices (VADs), MCS technology has advanced to provide more durable, efficient, and safer options for both shortand long-term support. This review outlines the historical development of mechanical assist devices, the types of available supports - ranging from intra-aortic balloon pumps and extracorporeal membrane oxygenation to implantable devices like HeartMate 3 - and their clinical indications and complications. Special attention is given to right ventricular dysfunction, thromboembolic and hemorrhagic complications, and infections, which remain major challenges in the management of patients with MCS devices.

In Brazil, despite the growing evidence supporting MCS in critically ill patients, access remains limited due to financial and systemic constraints. The review explores the current landscape of device availability in the country, national guidelines, cost-effectiveness data, and the impact of recent changes in transplant allocation criteria that prioritize patients receiving mechanical support. Notably, the approval of long-term VADs for destination therapy in the public health system in 2024 marks a significant milestone.

This review offers a comprehensive perspective on MCS utilization, highlighting both global advances and Brazil-specific challenges. By identifying gaps in access and proposing future directions, it advocates for expanded use of these life-saving technologies to improve survival and quality of life in advanced heart failure patients.

## INTRODUCTION

**Figure:**
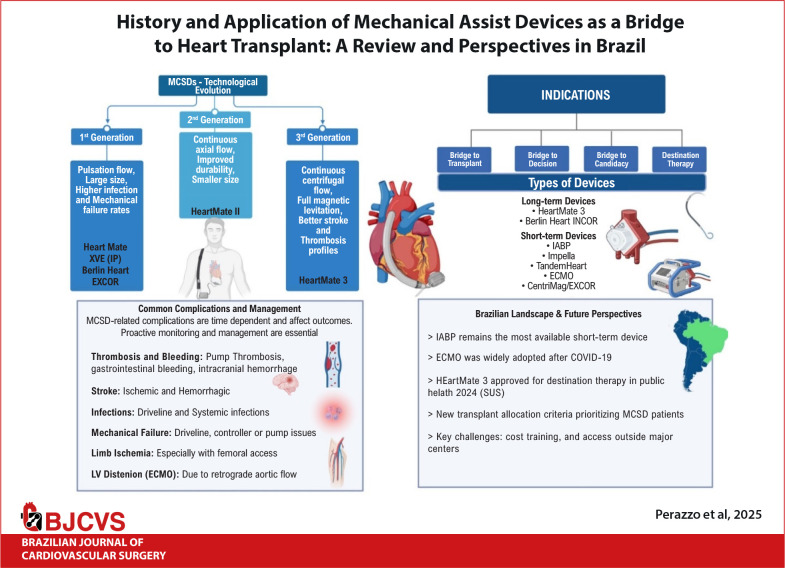

Heart transplantation (HTx) has long been the gold standard treatment for patients with end-stage heart failure (HF).^1^ However, the limited availability of donor organs and the associated waitlist mortality have prompted growing research in recent years into mechanical circulatory support (MCS) as an alternative solution for managing the challenges of restoring adequate hemodynamic function and ensuring a reasonable quality of life.

Over the decades, advancements in extracorporeal circulation have paved the way for the development of various devices aimed at assisting the treatment of HF in its advanced stages. This technological evolution has not only reshaped the management of end-stage HF globally but has also found its applications in Brazil, where the need for innovative solutions to address HF is equally critical.

MCS devices are vital tools in bridging patients to HTx. They promote hemodynamic stability and act as temporary support to extend survival by improving systemic perfusion until a suitable organ becomes available for transplantation. Furthermore, these devices can also provide long-term circulatory assistance as a destination therapy (DT) for those ineligibles for transplant.

In this context, the present article seeks to conduct a comprehensive review of the most significant MCS devices currently in use across the globe. Furthermore, this analysis extends to evaluating the availability, integration, and clinical outcomes of these technologies within Brazil’s healthcare system. By offering a detailed examination of the status of these devices in Brazil, this review aims to provide an insightful perspective on the current landscape of MCS in the country, highlighting both its achievements and the areas requiring further development and investment, a brief of this work is shown in Central Illustration.

History of mechanical assist devices

In the 1950s, Dr. John H. Gibbon introduced the “heart-lung machine” to support patients with perioperative complications and prolonged hemodynamic recovery.^2^ Building on this, growing interest in artificial circulation for HF patients led to the launch of a mission-oriented Artificial Heart Program, by the National Institutes of Health (NIH) in 1964, with legislative backing.^3 - 5^

In 1966, DeBakey et al.^6^ successfully used the first pneumatically driven paracorporeal left ventricular assist device (LVAD) to support a patient following cardiac surgery. The next year, Dr. Christiaan Barnard^7^ performed the first human HTx in Cape Town, and soon after, artificial ventricular technology began to be used as a bridge to transplant (BTT).

In 1969, Cooley et al.^8^ described the first use of a total artificial heart (TAH), as an idea of replacing the entire organ with an “artificial pump.” However, the device functioned for only a few days due to numerous complications, including infection, thrombosis, and hemolysis.

In the following decades, the focus was to develop mechanical pumps to assist the ventricles in providing adequate end-organ perfusion, reducing the risk of major thromboembolic complications, and allowing patients to survive until a compatible organ was available. For this vision to become a reality, the device needed to be rechargeable, easily transportable, and fully functional.

In 1984, DeVries et al.^9^ implanted the first TAH intended for DT, supporting the patient for 112 days.^9^ That same year, Portner et al.^10^ reported a successful case of BTT using a Novacor implantable electrical LVAD in a patient in cardiogenic shock due to ischemic heart disease.^10^

In 1994, the Food and Drug Administration (FDA) in the USA approved the first pneumatically driven LVAD as a BTT.^11^ Over the years, technological advancements have led to the integration of various mechanical systems as therapeutic options for shortand long-term artificial circulation in patients with advanced HF.

### Ventricular assistance device (VAD) - First generation (Pulsatile pumps)

The first-generation LVADs featured unidirectional artificial valves designed to mimic the pulsatile cardiac cycle, with diastolic filling and systolic emptying phases similar to the native heart.^12^ These devices could support patients with left, right, or biventricular failure (LVAD, RVAD, or BiVAD, respectively), and their primary objective was to offer long-term circulatory support, making them suitable as a BTT.^13^

These first-generation VADs, driven either pneumatically or electrically, included models like the Thoratec HeartMate IP (Implantable Pneumatic), VE (Vented Electric), XVE (Extended Vented Electric), and the Berlin Heart EXCOR.^14^ The HeartMate IP became the first LVAD to receive FDA approval in 1994. Clinical trials for the HeartMate VE began in 1992, and it was approved for DT in 2003, following the positive results of the Randomized Evaluation of Mechanical Assistance for the Treatment of Congestive HF (REMATCH) trial. In this trial, the interventional group demonstrated improved survival compared to medical therapy.^15^ An enhanced version of the original HeartMate, the HeartMate XVE, further improved 1-year survival rates in DT patients, 61% versus 52% in REMATCH.^16^ However, despite the survival benefits, many patients implanted with these first-generation pulsatile devices experienced significant adverse events, as infections, ischemic and hemorrhagic neurological injuries, and pump failure. The HeartMate VE (known as HeartMate I) and XVE were both pulsatile flow devices powered by electric motors.

### VAD - Second generation (continuous flow)

The second-generation pumps were much smaller than first-generations ones and featured a single internal rotor with a rotary extra-pericardial pump technology, and a continuous flow. In 1998, the second-generation VAD era began with the clinical use of the DeBakey VAD, a compact axial flow pump system.^17^ In 2001, Thoratec introduced the HeartMate II, which became the most widely implanted and studied LVAD of its time. Smaller and lighter than the original HeartMate XVE, it was approved for use in Europe and the USA in 2005, by the FDA as a BTT in 2008, and for DT in 2010.^2^ The HM2 was an axial-flow device designed with textured titanium lined internal surfaces contacting blood, in the attempt to minimize thrombosis. For eight years, from 2009-2017, it was the main LVAD implanted worldwide.^18^ In a trial comparing pulsatile devices with the newer generation of continuous-flow devices, a superiority of the latter was demonstrated, regarding both durability and neurological outcomes.^17^ Improvements in survival were observed with the growing expertise on both surgical technique and preand post-operative management of these patients.18 Nevertheless, despite such better results, and the reduction in severe adverse effects, it had still significant morbidity and mortality in comparison to HTx.^16 - 18^

### VAD - Third generation

Third-generation VADs have achieved significant advancements by reducing friction to minimize thrombosis within the continuous-flow pump and decreasing size to facilitate minimally invasive implantation techniques. The main examples are the HeartMate 3 and HeartWare Ventricular Assist Device (HVAD) - nowadays not commercially available, implanted directly into the left ventricle, in contrast with the second generation VADs, which are implanted extrapericadially. The HVAD employed a centrifugal impeller with hybrid magnetic/hydrodynamic suspension technology to reduce friction, while the HeartMate 3 features fully magnetic levitation.^19 - 21^

The MOMENTUM 3 trial compared the HeartMate 3 (centrifugal pump), with the HeartMate II (axial pump), in terms of outcomes for BTT and DT.^22^ The study involved 366 patients and found that 77.9% of those with the HeartMate 3 survived without disabling stroke or reoperation over a two-year follow-up, compared to 56.4% in the HeartMate II group.^3^ The HeartMate 3 also showed lower rates of pump thrombosis and ischemic stroke than the HeartMate II. There were no significant differences in sepsis, driveline infection, bleeding, right HF, arrhythmia, respiratory failure, renal dysfunction, hemolysis not associated to pump thrombosis, or hepatic dysfunction.

For five years, from 2012 to 2017, the HVAD pump was commonly used, also in children based on its smaller size compared to HeartMate 3. However, it was recalled by the FDA, and Medtronic ceased distribution in June 2021 due to increased risks of neurological events and mortality, associated with the internal pump and its ability to restart if it stopped.^3^ A study by The Society of Thoracic Surgery showed significantly higher mortality associated with hybrid levitation LVADs compared with fully magnetic levitation, with a survival at one year of 88% versus 79%.^23^ Nonetheless the risk associated with HVAD and HeartMate 3 exchange surpassed the risk of maintaining the HVAD with frequent monitoring.

### Types of mechanical assist devices

Mechanical assist devices can be classified based on duration (short or long-term), assisted ventricle (right ventricle, left ventricle, or biventricular), position relative to the patient (paracorporeal or implantable), and insertion technique (percutaneous, dissection, or surgical). Table 1 shows mechanical assist devices available in Brazil.

**Table 1:** Mechanical assist devices available in Brazil

SHORT-TERM DEVICES											LONG-TERM DEVICES	
		IABP^*^	TandemHeart	Impella				CentriMag^*^	Berlin Heart EXCOR^*^	ECMO venoarterial^*^	HeartMate 2^*^	HeartMate 3^*^
				CP^*^	5.0^*^	LD^*^	RP					
Ventricular assistance		LV					RV	LV and/or RV		LV and RV	LV	
Implantation		Percutaneous			Dissection	Percutaneous		Surgical		Percutaneous / surgical	Surgical	
Mechanism	Flow	Counterpulsation in aorta artery	Centrifugal	Axial				Centrífuga	Pulsátil	Centrífuga	Axial	Centrífuga
	Inflow		Left atrium (transseptal through the right atrium)	Left ventricle			Right atrium	VAD-R: right atrium VAD-L: left atrium or apex of the left ventricle		Right atrium	Apex of the left ventricle	
	Outflow		Femoral artery	Aorta artery			Pulmonary artery	VAD-R: Pulmonary artery VAD-L: Aorta artery		Aorta artery	Aorta artery	
Assistance time		Up to 30 days	Up to 30 days	Up to 7 days				Up to 30 days	Up to 90 days	Up to 20 days	-	
Maximal flow		0,5-1L/min	4 L/min	3,7 L/min	5 L/min	5 L/min	4 L/min	< 10 L/min	< 8 L/min	>4,5 L/min	-	
		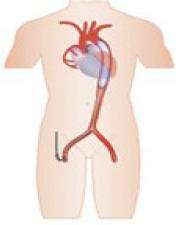	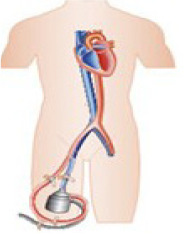	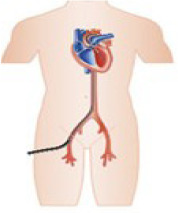			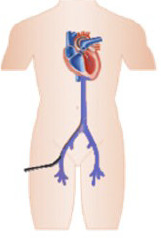	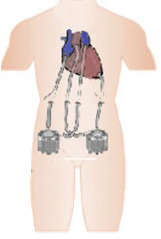	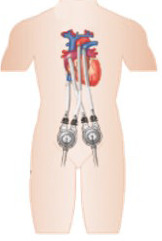	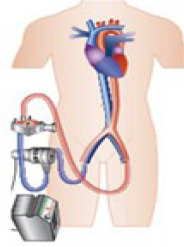	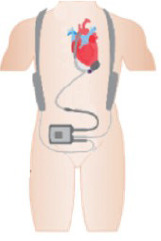	

ECMO: extracorporeal membrane oxygenation; IABP: Intra-aortic balloon pump RV: right ventricle; LV: left ventricle

### Short-term devices

Short-term devices are defined by their limited duration of use (dependent on the device) and currently include the intra-aortic balloon pump (IABP), extracorporeal membrane oxygenation (ECMO), TandemHeart, Impella CP, Impella 5.0, Impella RP, and CentriMag. The HeartMate is the primary long-term device currently available.^24 - 26^

• Intra-Aortic Balloon Pump: The IABP is the most widely available and used device in Brazil. It is inserted percutaneously via the femoral or subclavian artery, working with an aortic balloon inflating during diastole to increase coronary perfusion and deflating during systole to reduce left ventricular (LV) afterload. It offers hemodynamic support of approximately 0.5-1L/min for the left ventricle.^27 - 29^

• Impella: Also inserted percutaneously, Impella offers various models with a continuous axial flow pump, inserted through the femoral or axillary arteries into the left ventricle (or via the femoral vein into the right ventricle), providing hemodynamic support based on the selected model. For LV support, Impella CP delivers 3.7L/min, and Impella 5.0 provides 5L/min. Impella RP is designed for right ventricular support, offering up to 4L/min. In Brazil, available models include Impella CP, Impella 5.0, and Impella LD.^30 , 31^

• TandemHeart: This device is implanted through the femoral vessels, possibly percutaneously. It involves a draining cannula implanted from the femoral vein up to the left atrium through a transseptal atrial puncture and a perfusion cannula inserted into the femoral artery. Its purpose is to drain blood from the left atrium and to pump the already oxygenated blood into the iliofemoral arterial system, providing around 4L/min of support for the left ventricle.^32 , 33^

•CentriMag and EXCOR: Both paracorporeal devices are available in Brazil. CentriMag is a continuous centrifugal flow device with a free-floating contact-free magnetically levitated rotor. It is surgically implanted for left or right ventricular support.EXCOR, another paracorporeal device, delivers pulsatile flow and can support both ventricles, offering 8L/min of flow.^34 , 35^ Both devices are implanted through thoracotomy and pump blood from the right atrium to the pulmonary artery when supporting the right ventricle and from the left atrium or LV apex to the ascending aorta when supporting the left ventricle, providing up to 10L/min of flow. While the CentriMag has an approved duration of use of 30 days, the EXCOR can be used for longer periods of time, being considered a long-term pediatric device in the USA.^36^

• Extracorporeal Membrane Oxygenation: ECMO is the mechanical assist device of choice to multiple configurations for single ventricle, biventricular or respiratory support. It can be implanted percutaneously or via surgical dissection, either centrally or peripherally, and is classified as veno-arterial (V-A) or veno-venous (V-V). V-A ECMO provides both circulatory and respiratory support, while V-V ECMO only provides respiratory support. The flow can exceed 4.5L/min, adjusted according to hemodynamic needs.^37 - 40^

### Long-term devices

The most widely used long-term device in Brazil is the HeartMate 3. This third-generation device, following the HeartMate 1 and 2, operates using continuous centrifugal flow with magnetic levitation, providing LV support with a reduced complication rate compared to previous models.^41^

### Clinical indications

Circulatory assist devices, whether short or medium-term, can be utilized in situations requiring immediate hemodynamic support due to the high risk of death from HF. These devices can be temporarily employed as a BTT, for cardiac recovery, or as a bridge to decision when neurological prognosis is uncertain, or when there is a fine balance between increased survival and compromised quality of life.^42 , 43^

There is no exact definition of when to initiate MCS, but in patients with cardiogenic shock and persistent hypoperfusion despite pharmacological optimization of preor afterload (SCAI C, D, or E / INTERMACS 2 or 1), early initiation may mitigate the consequences of systemic hypoperfusion. ^[Bibr r44] 44- 46^

The ideal outcome involves balancing the level of hemodynamic support offered with the risk of complications. The choice of support involves the clinical and laboratory phenotype of the patient and the care goals. The experience of the team and institution should also be considered.^37^

The primary clinical indication for MCS is in the setting of cardiogenic shock, particularly in patients with acutely decompensated chronic HF. The decision to use it should be based on clinical, laboratory, and hemodynamic parameters, evidenced by sustained hypotension (systolic blood pressure, SBP <90mmHg and/or mean arterial pressure, MAP <65mmHg) with a cardiac index ≤ 2.2 L/min/m^2^, pulmonary artery occlusion pressure ≥ 15mmHg, and markers of systemic hypoperfusion (urine output <30mL/h, altered level of consciousness, cold extremities, and lactate >2mmol/L).^47^ Table 2 summarizes clinical indications and rationale behind MCS devices.

**Table 2:** Clinical indications and rationale for mechanical circulatory support devices

Clinical Indication	Rationale
Cardiogenic shock	Hemodynamics refractory to pharmacotherapy, acute myocardial infarction or heart failure related
Advanced heart failure	Preoperative optimization

Bridge to recovery, decision or durable therapy. Adapted from: Salter et al.^81^

In patients with contraindications (fixed pulmonary hypertension or sensitized immunological panel) or those who do not wish to undergo HTx, long-term devices have been used as bridge to candidacy or as DT. The development of modern devices, such as the HeartMate 3, with a lower incidence of complications, has proven ideal, especially for this patient profile.^48^

The selection of MCS should be integrated with the patient’s needs and the technical capacity of the service, as shown in Table 3 . The contraindications inherent to each device should also be considered, as described in Table 4 .

**Table 3:** Patient-tailored considerations for mechanical circulatory support device selection

Patient Needs	Technical Capabilities
Hemodynamic intensity deficit	Complication risk
Uni/biventricular Failure	Operator expertise
Therapeutic Goal	Institutional experience

Adapted from: Upadhyay et al.^82^

**Table 4:** Comparison of mechanical circulatory support devices

Device	Level of cardiac output support	Contraindications
Intra-aortic balloon pump	+/-	Moderate-to-severe aortic valve insufficiency, severe peripheral vascular disease, aortic dissection, aortic aneurysm
Microaxial flow pump (Impella CP)	++	Moderate-to-severe aortic valve insufficiency, left ventricular thrombus, mechanical aortic valve, severe peripheral vascular disease, aortic dissection
CentriMag (surgical)	++++	Complications of sternotomy or thoracotomy, bleeding, stroke
ECMO V-A	++++	Limb ischaemia, pulmonary oedema, intracardiac thrombus, stroke.
HeartMate 3	++++	Right ventricular dysfunction

Adapted from: Salter et al.^81^

### Clinical practice

MCS devices are associated with various complications that are generally time-dependent and that can significantly impact prognosis. These complications must be rigorously monitored and managed and most commonly occur within the first 90 days post-implant.49 Below are the key complications related to the use of MCS devices.

### Mechanical complications

Mechanical complications are inherent to the use of any circulatory support device, especially with prolonged dependency. These issues can arise from device component failures, improper placement, or structural wear due to continuous use.

• Device failure: All MCS devices can experience malfunctions that compromise circulatory support effectiveness. For instance, IABP failures can occur due to improper positioning, reducing the efficacy of counter-pulsation or causing arterial injuries.^49 , 50^ With the Impella, issues may include catheter fracture, myocardial perforation, and rotary system failure.^51^

• Mechanical wear: Prolonged use of devices such as LVAD can lead to wear of internal components and misalignment of rotors, compromising adequate blood flow. On the other hand, the most common technical issues with durable MCS devices are related to external components, including driveline rupture, controller and battery changes. These mechanical failures may require device revision or replacement.^52^ In case of LVAD as BTT, mechanical failure may represent an indication to prioritization of the patient on the HT waitlist, depending on the severity of the complication.

### Limb Ischemia

Limb ischemia is a complication particularly associated with devices requiring femoral access, such as the IABP, Impella, and V-A ECMO.

• Causes: The insertion of large-bore cannulas in MCS devices can impede distal blood flow, leading to lower limb ischemia. Studies report that this complication occurs in up to 10% of patients with V-A ECMO due to prolonged use of large-diameter femoral cannulas.^46^ Impella use has also shown a high rate of limb ischemia,^52^ with studies showing 0,07 - 10% reported incidence.^53^

• Prevention: Measures such as using ultrasound to guide vascular puncture and employing smaller cannulas can reduce risk. In many cases, the placement of distal perfusion cannulas beyond the main insertion site is necessary to maintain adequate limb blood flow and prevent severe ischemia.^54^

### Infections

Infections are among the most frequent complications in long-term MCS, such as LVADs - the 2020 INTERMACS registry report shows 41% of infection at one year post implantation.^49^ They can present as insertion site infections, systemic involvement, or infections related to external components.

• Insertion site, driveline and pump infections: The LVAD driveline, which crosses the skin, is a common entry point for pathogens and is particularly susceptible to chronic infections. Infection rates at the driveline site range from 20% to 40%, making this one of the leading causes of hospitalization and morbidity.^55 - 57^ Pump infection in BTT patients is an indication for urgent transplant.

• Localized and systemic Infections: Infections can progress to sepsis, especially in immunosuppressed or debilitated patients, significantly increasing mortality risk. Patients with implantable devices are frequently exposed to infections from Staphylococcus aureus and Pseudomonas aeruginosa, typical pathogens in hospital-acquired infections.^46^ LVAD patients have especially increased susceptibility for LVAD-related related infections, such as endocarditis and mediastinitis, which can occur in patients not on MCSDs but are more commonly observed in LVAD recipients.^49^

### Thrombosis and Hemorrhage

Anticoagulation is essential for preventing thrombosis in MCSDs; however, it also significantly increases the risk of hemorrhagic complications.

• Thrombosis: Thrombus formation can occur in both shortand long-term devices. In LVADs, blood stasis in the left ventricle, combined with prolonged contact between blood and non-biological surfaces, can lead to intracavitary thrombosis, increasing the risk of systemic embolism.^51 , 54^ When comparing second and third generation LVADs it is important to note that the MOMENTUM 3 trial showed significantly lower pump thrombosis incidence on third generation devices, dropping from 8-10% rates at one year for the HVAD and HeartMate2 to 1.4% for the HeartMate3.^49^ In temporary MCS, the oxygenator is the component more prone to thrombosis.^37^

• Hemorrhage: Systemic anticoagulation, necessary to prevent thrombosis in devices such as ECMO, significantly raises the risk of bleeding, with intracranial hemorrhages being the most feared due to their catastrophic potential. Studies indicate that 5% to 10% of ECMO patients develop severe hemorrhagic events.^55^ For LVAD recipients, hemorrhagic complications are most frequently related to hematologic alterations related to the continuous flow resulting in acquired von Willebrand Factor deficiency and platelet disfunction. In general, studies show that approximately 33% of patients experience major bleedings, 50% of which occurs in the gastrointestinal tract.^49^

• Balancing anticoagulation and bleeding risk: Anticoagulation management requires constant monitoring, using parameters such as activated clotting time, activated partial thromboplastin time (aPTT) and international normalized ratio (INR). Anticoagulation strategies can be adjusted according to the patient’s thrombotic and hemorrhagic risk^57^ findings for the MOMENTUM 3 and MAGNETUM 1 trials tend support lower intensity anticoagulation in HeartMate3 patients.^49^

### Stroke

Stroke is a critical complication associated with MCS devices and is one of the leading causes of morbidity and mortality in these patients. Strokes can occur in both ischemic and hemorrhagic forms, resulting from the need for anticoagulation, hemodynamic alterations, or the presence of non-biological surfaces that promote thrombus formation. The incidence of stroke varies by device type and patient profile. In patients with LVADs, the ischemic stroke rate is approximately 10% to 20%, while hemorrhagic stroke is less common but potentially more lethal. The most critical time is the early post operative period, which requires close monitoring: 50% of stokes occur within the first seven days and 70% of those within the first 48 hours after surgery, reducing significantly the risk at two months after the procedure.^58^ It is important to highlight the significant reduction in stroke risk for HeartMate3 patients, who have a 77% lower risk compared to those with second generation LVADs. In ECMO patients, cerebral hemorrhagic events occur in about 5% to 10% of cases, with a steep increase in incidence after 10 days of support.^37 - 39^

• Ischemic Stroke: This is the most common type of stroke associated with LVADs and other devices, caused by thrombus formation in the left ventricle or on the device’s non-biological surfaces. Other related risk factors include atrial fibrillation, diabetes and reduced anticoagulation due to hemorrhagic events.^49^ These thrombi can embolize into the arterial system, leading to cerebral infarctions. Blood stasis in the left ventricle, especially in patients with inadequate anticoagulation or severe ventricular dysfunction, increases the risk of thrombogenesis.^51 , 54 , 59^

• Hemorrhagic Stroke: Anticoagulants, necessary to prevent thrombosis in ECMO, LVAD, or Impella patients, increase the risk of severe bleeding, such as intracranial hemorrhage. This type of stroke is frequently related to coagulopathy induced by the MCS itself, especially in ECMO, where continuous anticoagulation is critical to prevent thrombi formation in the circuit.^49 , 50 , 55^ Studies show that infection and hypertension with mean arterial pressure ≥ 90mmHg are also associated with increased hemorrhagic stroke risk.^49^

### Right ventricular dysfunction in long-term devices

Right ventricular dysfunction (RVD) is a frequent complication in patients with long-term devices like LVAD, occurring in up to 30% of cases.^55 , 59^

• Etiology: After LVAD implantation, increased venous return to the right side of the heart, combined with the right ventricle’s inability to cope with the additional load, and the leftward shifting of the ventricular septum can lead to severe RVD. This problem is exacerbated in patients with pre-existing RVD or pulmonary hypertension,^52^ which per se may be a contraindication to LVAD implantation.

• Clinical impact: RVD can result in systemic venous congestion, leading to liver and kidney dysfunction. Moreover, improper LV filling due to RVD might lead to improper LVAD functioning with significantly decrease in cardiac output. Mortality associated with RVD in LVAD patients is significantly elevated, making it one of the leading causes of post-implantation complications.^54^

• Management: Treatment includes optimizing preload and using inotropic support for the RV. In severe cases, BiVADs may be required.^55^

### Left ventricular distension in ECMO

LV distension is a common and severe complication in patients on V-A ECMO.

• Causes: V-A ECMO increases LV afterload by delivering retrograde flow into the aorta, which can prevent effective ventricular emptying, also interfering with aortic valve opening, leading to LV distension and subsequent pulmonary congestion. This complication is more common in patients with mitral insufficiency or low ventricular compliance.^55 , 59^

• Consequences: LV distension can cause severe pulmonary congestion, increase the risk of thrombus formation within the ventricle, and impair cardiac recovery. If left untreated, it can exacerbate LV failure and compromise ECMO’s effectiveness.^54^ As a matter of fact, various unloading systems and configurations have been described to prevent LV distension in V-A ECMO, such as pulmonary artery cannula, apical venting or combined adoption of Impella (i.e. ECPELLA).

### Guidelines for the use of mechanical circulatory support devices

Leading international societies such as the Brazilian Society of Cardiology (SBC), International Society for Heart and Lung Transplantation (ISHLT), European Society of Cardiology (ESC), and American Heart Association (AHA)/American College of Cardiology (ACC) have published evidence-based guidelines to assist in decision-making regarding the use of MCS devices. These guidelines provide recommendations on indications, contraindications, and management of both shortand long-term devices, including their use as a BTT, DT, and in cases of cardiogenic shock. Main recommendations of these guidelines are described in Table 5 .^57 , 60 - 63^

**Table 5:** Indications for mechanical circulatory support devices according to different societies

Indication	Brazilian society of Cardiology (SBC)	International Society for Heart and Lung Transplantation (ISHLT)	European Society of Cardiology (ESC)	American Heart Association (AHA) / American College of Cardiology (ACC)
Bridge to Transplantation	Recommended for patients with end-stage heart failure on the heart transplant waiting list (Class I Recommendation, Level of Evidence B)^60^	Recommended for hemodynamic stabilization in patients awaiting heart transplantation (Class I Recommendation, Level of Evidence A)^57 , 61^	Should be carefully evaluated through a multidisciplinary approach^62^	Recommended for patients with advanced heart failure refractory to medical treatment (Class I Recommendation, Level of Evidence B)^63^
Bridge to Recovery	Recommended in cases of potentially reversible acute heart failure, such as myocarditis, until ventricular function recovers (Class IIa Recommendation, Level of Evidence C)^60^			
Bridge to Decision	Recommended for patients with uncertain prognosis, as a temporary solution until a definitive clinical decision (Class IIa Recommendation, Level of Evidence C)^60^	Recommended in unstable patients whose clinical viability is still being assessed (Class IIa Recommendation, Level of Evidence B)^57 , 61^		
Destination Therapy	Recommended for patients with left ventricular heart failure who are ineligible for transplant, as of December 2024^60^	Recommended to improve survival and quality of life (Class I Recommendation, Level of Evidence B)^57 , 61^	Should be carefully evaluated through a multidisciplinary approach^60^	Recommended to improve survival (Class I Recommendation, Level of Evidence A)^63^
Refractory Cardiogenic Shock / Advanced Chronic Heart Failure			Short-term devices are recommended in patients with cardiogenic shock unresponsive to medical therapy (Class I Recommendation, Level of Evidence B) Long-term devices are recommended in patients with severe and irreversible ventricular dysfunction (Class I Recommendation, Level of Evidence B)^62^	
Temporary Support for High Risk Procedures				Short-term devices are recommended in high-risk patients requiring invasive procedures like revascularization (Class IIa Recommendation, Level of Evidence B)^63^

The guidelines also establish contraindications for the use of mechanical VADs. The main absolute contraindications include:^57 , 60 - 63^

• Severe Aortic Insufficiency: Significant aortic insufficiency prevents the effective operation of the device, especially in long-term devices like LVAD, as regurgitation through the aortic valve can cause LV volume overload, increasing the risk of distension and HF.

• Intracavitary thrombi: The presence of thrombi in the heart chambers is a major contraindication, as using mechanical devices like Impella or LVAD can increase the risk of systemic embolism, leading to potentially fatal events such as stroke or peripheral embolism.

• Aortic dissection: Patients with aortic dissection are contraindicated for short term MCS devices due to the risk of exacerbating the aortic lesion and rupture during assisted circulation. On the other hand, in some cases of post cardiotomy, the ECMO can be used in dissection patients.^64^

In addition to these absolute contraindications, the guidelines also list relative contraindications depending on the patient’s clinical status and response to therapy. Patients with active sepsis or uncontrolled infections are contraindicated for long-term devices, as the risk of mortality significantly increases due to infection spread. Likewise, irreversible multi-organ dysfunction, particularly involving the liver and kidneys, is considered a major contraindication, as it compromises the potential benefits of the device and increases perioperative complications and short-term mortality.

In the context of short-term devices, such as V-A ECMO, ESC and AHA/ACC guidelines emphasize that patients with severe LV failure and inability to decompress the ventricle may experience worsened cardiac function due to ECMO-induced retrograde flow. Moreover, pre-existing coagulopathy or a high risk of bleeding contraindicate ECMO use due to the need for continuous anticoagulation and the risk of severe hemorrhagic complications, such as intracranial hemorrhage.

Finally, all guidelines highlight the importance of a rigorous multidisciplinary evaluation to identify these contraindications before deciding to implant devices. Preoperative evaluation should include not only hemodynamic parameters and ventricular function but also a thorough examination of comorbidities and the patient’s overall condition to avoid implantation in high-risk scenarios. Careful selection is crucial to ensure that patients benefit with minimal complications, optimizing both shortand long-term outcomes.

### Right ventricular dysfunction in LVAD patients

RVD is a well-documented and critical complication in patients receiving LVADs, and all guidelines emphasize the importance of careful assessment of right ventricular function prior to device implantation. The ISHLT, ESC, and AHA/ACC guidelines classify RVD as a significant limiting factor in the implantation of LVADs.

• The ISHLT emphasizes that up to 30% of patients who receive an LVAD develop significant RVD after implantation, which increases mortality and prolongs hospitalization. In these cases, the use of biventricular support (BiVAD) or temporary right VADs, such as the Impella RP or RVAD, may need to be considered.^57^

• The ESC guidelines highlight that pre-existing RVD is one of the strongest predictors of adverse outcomes following LVAD implantation and recommend that all patients undergo evaluation of RV function before implantation (Class I, Level of Evidence B). In patients at high risk of RV dysfunction, consideration should be given to using BiVAD support or temporary RV devices to minimize systemic congestion and improve pulmonary flow.^62^

• The AHA/ACC guidelines also emphasize that RVD post-LVAD implantation is a severe complication, often resulting from the volume overload imposed on the right ventricle due to the increased venous return to the right heart following LV decompression. Management of RVD includes inotropic support, optimization of preload, and, in more severe cases, the use of temporary RV support devices (Class IIa, Level of Evidence B).^63^

All the guidelines are unanimous in stressing that pre-implant assessment of RV function is critical for predicting the need for additional RV support. Patients with severe RVD and significant systemic congestion may not be suitable candidates for isolated LVAD implantation, and biventricular support should be considered in these cases. Additionally, continuous monitoring of right ventricular function post-implant is essential, with early interventions to prevent hemodynamic complications and ventricular failure. In patients with borderline RV function, it has been described a staged procedure where trial LV support either via Impella or via paracorporeal LVAD is attempted to evaluate the consequent RV response. Such approach might provide further information about durable LVAD implantation eligibility.

### Overview in Brazil

The use of MCS devices is a substantial element in the treatment of cardiogenic shock approved in Brazil. Although included in both international and Brazilian guidelines for this condition, the current landscape reveals a lack of financial support and encouragement from healthcare agencies for their use in the country.^65^ Despite advances in therapeutic options, patients often have no option for HTx or circulatory support devices.^66^

There is limited available data regarding the usage and cost of this technology at a national level, directing evaluation based on international scenarios. Cost-effectiveness studies compared Impella and IABP and showed an incremental cost effectiveness ratio varying from €38,069/$52,063 e €31,727/$43,390 per year of life saved and adjusted for quality.^67^ This is especially relevant considering that 53.8% of HTx patients from 2013 to 2024 (528 consecutive heart transplants) in the largest Brazilian heart transplant center used IABP. Furthermore, other evidence estimates an average value between $85,025 a $1,257,946 US Canadian dollars per year of life saved and adjusted for quality, among the various assistance devices.^68 , 69^ In addition, it is estimated that 24% of patients selected for transplantation die while still on the waiting list, whereas these devices are responsible for survival rates exceeding 70% in one year, creating the possibility of transplantation for many assisted patients.^70^

The first experience with MCS devices in the country is reported in 1994, used in a Chagas patient as a bridge therapy for successful transplantation. The equipment was developed by the bioengineering service of the Heart Institute (InCor) of the General Hospital of University of São Paulo Medical School (HCFMUSP, São Paulo, Brazil). Meanwhile, the first reported case of a patient being discharged after implanting an implantable ventricular assist device and subsequent transplantation occurred in 2012 (Berlin Heart INCOR).^71^

According to approval and registration by the National Health Surveillance Agency (Anvisa), the main temporary devices currently available in Brazil include IABP counterpulsation; ECMO; TandemHeart; Impella (CP); CentriMag and Berlin Heart EXCOR. As for long-term devices available in Brazil, HeartMate III and Berlin Heart INCOR are the main ones.

Counter-pulsation systems were experimentally described starting in 1952 by Adrian Kantrowitz, with the development of the IABP by Moulopoulos in the 1960s. Despite evidence of the superiority of other temporary devices, the IABP remains the most accessible and easy-to-implant device, with lower cost and fewer complications when compared to others. It enables implantation in healthcare facilities without cardiac surgery or hemodynamic services available, facilitating its broader diffusion and remaining the most widely used circulatory support device in the country. Some of the available brands in the country include Maquet, Getinge, and Arrow/Teleflex, with the equipment device average found about R$60,000,00 or R$172,000.00. When analyzed the price related with the procedure, the estimated cost is around R$3,700,00 and R$11,588.72.^72^

Finally, despite ECMO use initiate in the 1970s, in Brazil, it was only in 2016 that a formal recommendation was made with the Mechanical Circulatory Support Guidelines of the SBC and the Federal Council of Medicine (CFM - opinion 42/2017), no longer considering it as an experimental procedure. ECMO is now widely used as a BTT or recovery. Currently, 22 centers (across 11 cities) are accredited in the country for its use, authorized by the Extracorporeal Life Support Organization (ELSO LATINO-AMERICA),^36 , 72^ an institution involved in the care and training for the use of this device. The country’s most populous capitals have the equipment, especially in São Paulo, with 10 certified hospitals.^73^ This number may be considered insufficient compared to the demand and volume of transplants performed when compared to other countries, such as the U.S., where 48 cities and hundreds of centers have availability.

There has been greater dissemination of MCS devices after the COVID-19 pandemic (2020) due to the high number of patients with respiratory failure caused by the Sars-CoV-2 infection. This movement allowed for the expansion of equipment availability in services across the country, including its use as a circulatory support device. However, it remains a high-cost therapy with low accessibility outside referral centers. The estimated cost per patient ranges from 55,000 to 155,000 Brazilian reals, varying by manufacturer, but with evidence of cost-effectiveness in the literature.^74^ The registered companies authorized to commercialize ECMO in Brazil include Eurosets, Maquet, Nipro, and Sorin.^75^

Recent adjustments in HTx allocation criteria in Brazil have significantly influenced outcomes, particularly for patients requiring MCS. Inspired by changes introduced by the United Network for Organ Sharing (UNOS) in the U.S., these modifications were designed to reduce waiting list mortality and ensure that the most critically ill patients receive priority for HTx. In 2020, the state of São Paulo adopted these revised prioritization levels, which have notably benefited patients supported by ECMO or IABP.^76 - 79^

The revised system established three distinct prioritization levels:

1. First condition (highest priority): Patients requiring acute retransplantation within 30 days post-transplant, those on V-A ECMO, or those requiring shortto medium-term mechanical circulatory assistance.

2. Second condition: Patients supported by an IABP, those with malfunctioning mechanical circulatory devices, or patients on artificial ventilation due to HF decompensation.

3. Third condition: Patients in cardiogenic shock requiring one or more inotropes for more than six months, patients on long-term MCS with complications, patients with ischemic cardiomyopathy with refractory angina, and those with congenital heart disease. After six months of continuous prioritization in this group, patients are elevated to the second priority level, improving their chances for HTx.^77 - 79^

These changes have already shown positive impacts, particularly in reducing waiting times and mortality rates among ECMO-supported patients. Patients prioritized by inotropic therapy now benefit from automatic elevation in status after 180 days, leading to an increased likelihood of receiving a transplant. Early studies at major transplant centers, such as the Heart Institute (InCor), confirmed that these new criteria significantly improved outcomes for critically ill patients.^75 - 77^ While the initial results are promising, further research is required to assess the long-term effects of these changes across the country.

### Future perspectives

Despite considerable technological progress in VAD therapy over the last decades, the significant rate of complications and impaired quality-of-life are still significant challenges, making them far from optimal for patients with end-stage HF. Future VADs should target long-term survival rates comparable to HTx. Incremental improvements, such as enhanced blood-pump interfaces and impeller designs, aim to improve hemocompatibility. More ambitious goals include developing VADs that dynamically adjust flow and replicate natural pulsatile patterns. Fully implantable devices and machines with autonomous adaptation to physiological needs could optimize performance. All these advancements may eventually surpass the outcomes of HTx.

## Conclusion

The history and application of MCS devices, particularly as a BTT, have significantly evolved over the past few decades. In Brazil, however, despite the technological advancements in MCS, the access to these devices remains limited due to financial constraints and infrastructural challenges. The availability of MCS devices is dependent on the public healthcare system and supplementary healthcare, where private hospitals provide services to the public sector to satisfy the demand. Nevertheless, these devices remain critical in managing end-stage HF, especially in cases where HTx is delayed due to organ scarcity.

Mechanical assist devices have proven effective in improving patient outcomes, offering a vital solution for those awaiting HTx, with significant reduction in adverse events and improved quality of life due to the development of third generation devices. Yet, complications such as RVD, device thrombosis, and infection continue to pose challenges, highlighting the need for improved patient selection and postoperative care.

Brazil’s experience with these devices reflects global trends, with the first successful use of MCS in a Chagas disease patient in 1994 marking the country’s entry into this field. Additionally, recent revisions in HTx allocation criteria in São Paulo, influenced by international guidelines, have improved transplant outcomes by prioritizing patients with mechanical support. However, continuous research and investment are crucial to expand access to these life-saving technologies across the country and ensure the long-term sustainability of MCS therapies.

In conclusion, while MCS technologies offer substantial benefits for patients with advanced HF, Brazil must continue to address the systemic barriers that limit their use, expanding accessibility and optimizing patient management. Notably, the approval of LVADs as DT in the public healthcare sector as of December 2024 marks a significant milestone and is an example of innovative politics that will be vital to increase survival rates and quality of life of patients with end-stage HF.^80^

## Study association

This study is not associated with any thesis or dissertation work.
